# The scientific impact of the Sir Charles Bell Society in head and face medicine

**DOI:** 10.1186/1746-160X-3-24

**Published:** 2007-05-25

**Authors:** Rainer Laskawi, Jaqueline Diels, Paul Mulkens, Mihael Podvinec

**Affiliations:** 1 ENT-Department, Universitiy of Göttingen, Robert-Koch-Str. 40, D-37075 Göttingen, Germany; 2 Department of Orthopedics and Rehabilitation, University of Wisconsin Hospital and Clinics, Research Park Clinics, 621 Science Dr., Madison, WI 53711, USA; 3 Vaart NZ 96^e^, 9406 CN Assen, The Netherlands; 4 ENT-HNS-Department, Cantonal Hospital, Aarau, Switzerland

## Background

The "Sir Charles Bell Society (SCBS)"[1] is an international multi-disciplinary organization created at the "VII International Facial Nerve Symposium (Cologne 1992)", dedicated to collection, dissemination and interchange of ideas relating to the facial nerve, thus furthering cooperation and encouraging global friendships. The SCBS is a non-profit world-wide organization with headquarters in San Francisco, California, USA. The SCBS is open to all clinicians, pre-clinicians, researchers, residents in training etc. with professional and scientific interest in the facial nerve.

The SCBS began as an informal group in 1992 during the VIIth International Facial Nerve Symposium in Cologne. At this initial meeting, 74 attendees confirmed the need for a multinational approach. The original 74 attendees were to be considered as "Founding Members" and they drafted a Statement of Purpose: "The SCBS is to be an international forum for clinicians of all medical specialties and researchers in basic or clinical science who have a special interest in the facial nerve and its disorders. This unique international multi-disciplinary organization is dedicated to the collection, dissemination and exchange of ideas relating to the facial nerve and its diseases." Their first charge was to aid Professor Naoki Yanagihara in organizing the "VIIIth International Facial Nerve Symposium" to be held in Japan in 1997.

The SCBS was officially recognized June 23, 1993, incorporated in the state of California as a non-profit mutual benefit corporation organized under the non-profit mutual benefit corporation law meeting the criteria of: Internal Revenue Code Section 501 C (6) with FEDERAL Employee Identification Number: 94-320802 and California Corporate Number; 1917755 SCOBS.

Membership includes otolaryngologists, plastic-reconstructive surgeons, neurologists, neurosurgeons, basic science researchers, physiotherapists, audiologists, anatomists, psychologists, psychiatrists, radiologists, and internists. At present there are over 229 members from 32 countries.

The scientific impact is based on the interdisciplinary discussion of problems dealing with the facial nerve. An international meeting is held every four years in different countries. Main topics of discussion and international exchange are:

▶ Facial nerve regeneration following different kind of lesions

▶ Changes of the Central Nervous System following different kind of nerve lesions

▶ Surgical and conservative methods for treatment of facial nerve diseases

▶ Evaluation of grading systems.

An important aspect of the SCBS is that these problems are discussed on an interdisciplinary platform.

The last meeting of the SCBS was held in 2005 in Maastricht/Netherlands during the "Xth International Facial Nerve Symposium (Chairman: Hans Manni)" with election of Rainer Laskawi (Germany), President; Jacqueline Diels (USA), Secretary; Paul Mulkens (Netherlands), Treasurer, Mihael Podvinec (Switzerland) is Webmaster and Kedar Adour (USA) President-Emeritus. The next meeting will be held in Rome (Italy) in 2009.
